# Genetic Incompatibility Dampens Hybrid Fertility More Than Hybrid Viability: Yeast as a Case Study

**DOI:** 10.1371/journal.pone.0018341

**Published:** 2011-04-06

**Authors:** Meibo Xu, Xionglei He

**Affiliations:** State Key Laboratory of Bio-Control, College of Life Sciences, Sun Yat-sen University, Guangzhou, China; University College London, United Kingdom

## Abstract

Genetic incompatibility is believed to be the major cause of postzygotic reproductive isolation. Despite huge efforts seeking for speciation-related incompatibilities in the past several decades, a general understanding of how genetic incompatibility evolves in affecting hybrid fitness is not available, primarily due to the fact that the number of known incompatibilities is small. Instead of further mapping specific incompatible genes, in this paper we aimed to know the overall effects of incompatibility on fertility and viability, the two aspects of fitness, by examining 89 gametes produced by yeast *S. cerevisiae* - *S. paradoxus* F1 hybrids. Homozygous F2 hybrids formed by autodiploidization of F1 gametes were subject to tests for growth rate and sporulation efficiency. We observed much stronger defects in sporulation than in clonal growth for every single F2 hybrid strain, indicating that genetic incompatibility affects hybrid fertility more than hybrid viability in yeast. We related this finding in part to the fast-evolving nature of meiosis-related genes, and proposed that the generally low expression levels of these genes might be a cause of the observation.

## Introduction

Postzygotic reproductive isolation is a key step of forming new species, and the underlying genetic mechanisms can be multiple [1], [2], [3], [4]. The model of between-gene incompatibility proposed by Dobzhansky [5] and Muller [6] posits that independent evolution of two populations can generate alleles that are completely normal on native genetic background but deleterious on genetic background of the other population, rendering hybrid inviability or sterility. This is an appealing idea because it predicts that new species can evolve as a time-dependent by-product of geographical separation of populations, and has therefore received a lot of attention. Efforts in the past several decades have revealed a handful of genetic incompatibilities in organisms including fly [7], [8], [9], [10], nematode [11], mouse [12], yeast [13], and plants [14], [15]. Although the evolution of genetic incompatibility between two species is inevitable even if the speciation is not due to incompatibility, such incompatibilities identified from recently formed species are probable speciation-causing genes, and thus of great interest to people. Some common features were observed from these probable speciation genes [16]; for example, they tend to be fast evolving. However, a general understanding of how genetic incompatibility evolves in affecting the fitness of hybrids is still lacking. An organism's fitness is influenced by its ability to survive and develop/grow (viability), and its ability to reproduce (fertility). Available incompatibilities identified from different organisms show an equivocal pattern regarding their effects on the two components of fitness [16]. It is the aim of this study to systematically test whether genetic incompatibility affects hybrid fertility and hybrid viability differently.

**Figure 1:**
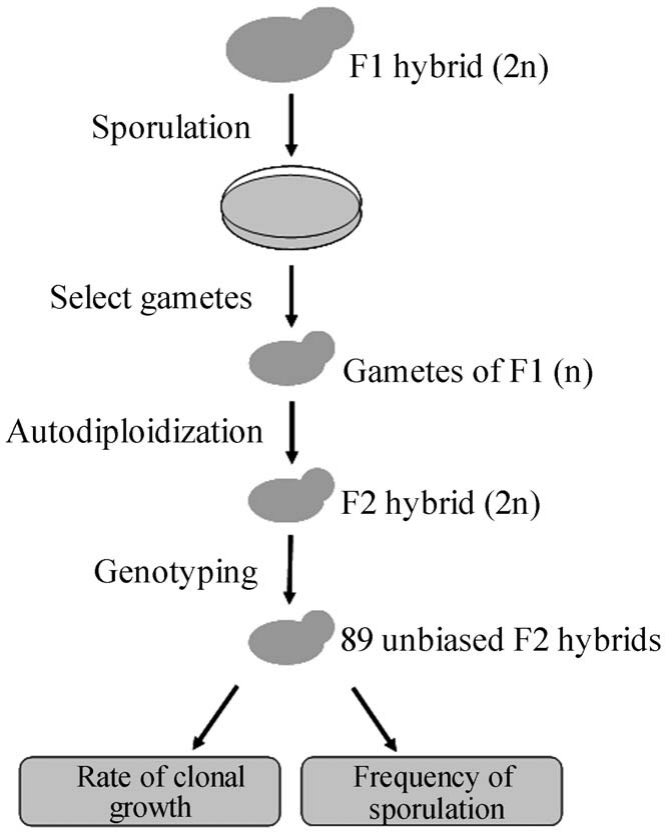
A schematic map of the experimental design.

It should be pointed out that the diploid F1 hybrids are not suitable for addressing the above question, because: 1) the heterozygous nature of F1 hybrid genome will mask the effects of recessive incompatibility, and 2) in addition to genetic incompatibility, some confounding factors such as chromosomal translocation and sequence divergence may also reduce F1 hybrid fertility by disturbing meiosis [17]. To overcome the two problems, homozygous F2 hybrids are needed. In addition, two species that are close enough to form a hybrid but are diverged sufficiently to have evolved a plenty of genetic incompatibilities are desired. In this paper, the hybrid of yeasts *Saccharomyces cerevisiae* (*Sc*) and *Sacchromyces paradoxu*s (*Sp*) was examined; the two *Saccharomyces* species split ∼10 million years ago, being ∼15% diverged in genome sequence [18]. We were fully aware that, given the significant sequence divergence, any specific incompatibility identified from the two yeasts is more likely to be the aftermath rather than the cause of speciation. This, however, still fits our interest in understanding how genetic incompatibility evolves between two separated populations. The haploid gametes of F1 hybrids underwent autodiploidization to form diploid homozygous F2 hybrids. Fertility and viability of the yeast were measured by the sporulation frequency and the clonal growth rate, respectively, for each of the F2 hybrids (Figure 1). We obtained strong evidence supporting that genetic incompatibility affects hybrid fertility more than hybrid viability in yeast.

## Results

The heterozygous F1 hybrid formed between *Sc* and *Sp* grows normally but produces only ∼1% viable gametes, while both parental species can produce 90–100% viable gametes [19]. Fortunately, the population of yeast cells is large enough, so sufficient F1 gametes were readily available. A total of 94 gametes were randomly selected. Genetic incompatibility could be a cause for the ∼99% inviable F1 gametes, through either preventing gamete formation or killing the gametes once formed. The incompatibility of preventing gamete formation in the heterozygous F1 hybrid, if present, must be dominant; however, a previous work has demonstrated that there is no dominant genetic incompatibility in the yeast hybrid [20]. The remaining concern was that genotypes with lethal incompatibility that kills gametes, if exist, can not be obtained, so the 94 gametes may contain ascertainment bias, resulting in underestimation of the effects of incompatibility on viability.

### Genotyping the 94 selected gametes using 93 markers

**Figure 2:**
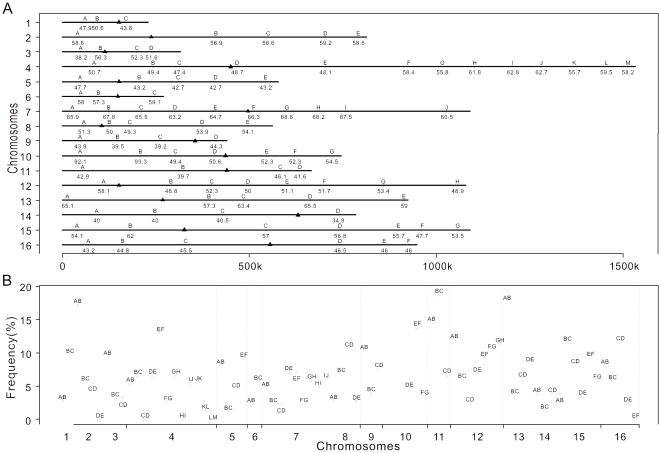
Marker position and the recombination rate between neighboring markers. (**A**) A schematic map of the 93 markers on 16 yeast chromosomes. The black triangle shows the position of centromere. The capital letter above the line is marker name, and the number under the line is the percentage of gametes being of *Sc* origin for the corresponding marker. Note that two markers 10A and 10B are of *Sc* origin in >90% of gametes, an observation that can not be reproduced in other *Sc−Sp* hybrids. (**B**) The recombination rate between neighboring markers is generally low. Three blocks (AB, BC, and CD) at the chromosome 10 were not included.

Ninety-three markers encompassing the whole yeast nuclear genome were designed to genotype the 94 gametes, to evaluate the potential ascertainment bias (Figure 2A; Table S1). We ignored the mitochondrial genome because the *Sc−Sp* F1 hybrid we used contains only *Sc* mitochondrion. Five gametes were excluded from further study because they show significant (*P*<10^−5^; Pearson correlation analysis) genotypic similarity to other gametes. Among the remaining 89 gametes, there are a similar number of *Sc* origins and *Sp* origins for all makers, with the exception of markers 10A and 10B that are of *Sc* origin in >90% of gametes (Figure 2A; Table S2). The result is unexpected because a previous study tested the whole chromosome 10 of *Sp* and found no incompatibility of this *Sp* chromosome with *Sc* genome [19]. To resolve this inconsistency, we constructed a new F1 hybrid using different *Sc* and *Sp* strains, and found that our above observation can not be reproduced (a total of 21 gametes were examined, and the marker 10B was from *Sc* in 9 gametes and from *Sp* in the other 12 gametes). The *Sp* strain, YDG749, used in our confirmation experiment is the same one used by the previous study [19], while the *Sp* genome of the original F1 hybrid was from YCM361. So, it is likely that a mutation occurred to an essential gene of the *Sp* locus when the original F1 hybrid was made or cultured in laboratory. We made no attempt to find the mutation from the ∼150 kb region and ignored this single suspicious locus in further study, because the primary goal of this work is to assess the overall effects of a mixture of unknown incompatibilities.

### No ascertainment bias was detected from the 89 gametes

It was proposed that sequence divergence between *Sc* and *Sp* suppresses recombination in the *Sc−Sp* F1 hybrid [4]. Indeed, despite the median distance of two neighboring markers being 103 kb, the median recombination rate between two markers is 5.7% (Figure 2B), much smaller than the rate of ∼1 recombination per 100 kb per meiosis in *Sc* [21]. The suppressed recombination helped us to identify the origin of a block between two markers with high confidence. For example, markers 1-A and 1-B are both from *Sp* in gamete 3, and both from *Sc* in gamete 6, so the 32 kb block between 1-A and 1-B is of *Sp* origin in the gamete 3, and of *Sc* origin in the gamete 6, each with a roughly estimated false discovery rate (FDR) of 0.07%, given that the recombination rate between 1-A and 1-B is 2.7% (two recombination events are needed when 1-A and 1-B are from the same yeast but the block 1-AB contains genes of different yeasts); the origin of block 1-AB will not be assigned for gametes in which 1-A and 1-B are from different species. Since we did not design markers for telomeres, the origins of two ends of a chromosome were based on the single closest marker.

**Figure 3:**
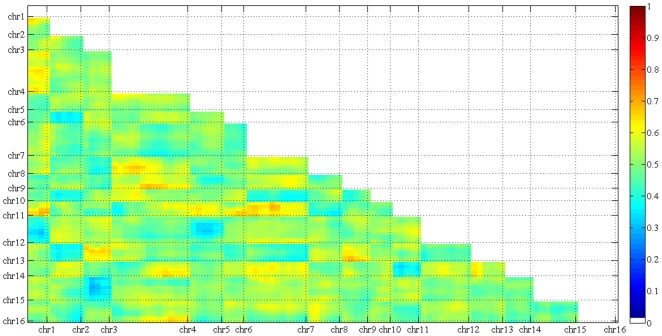
No between-chromosome two-locus incompatibility was detected. The fraction of heterogeneous combination ((*ScSp*+*SpSc*)/(*ScSc*+*ScSp*+*SpSc*+*SpSp*)) is shown for each of the 5111 block pairs. None is significantly different from 0.5, the expected value, at an FDR of 1%, as determined by Chi-square test. Note that the 4 blocks at the left arm of chromosome 10 were excluded from the analysis.

The 93 markers separate the yeast genome into 109 blocks. If there is lethal incompatibility between two loci, one would expect the absence of heterogeneous combination (i.e., two blocks are from different yeasts) between the corresponding blocks. Interestingly, we observed the absence of heterogeneous combination only for blocks on the same chromosomes, which is presumably due to the low recombination rate in the F1 hybrid [4]. Heterogeneous combination was observed for all between-chromosome block pairs; in fact, none of these block pairs showed significant under-representation of heterogeneous combination at an FDR of 1% (i.e., the frequency of heterogeneous combination is at least 26/89 = 29.2%, because with 89 strains an observed ratio of 25∶64 will be regarded as significant deviation from expectation with the Chi-square *P* = 0.007) (Figure 3). The same is true when one-way incompatibility was tested (data not shown). The result is in well line with a previous study in which a whole *Sc* chromosome was replaced by its *Sp* homologous chromosome and no lethal or severe phenotype was observed for all 9 tested chromosomes (amount to ∼43% of the yeast genome). Thus, it seems unlikely that lethal or nearly-lethal genetic incompatibility affecting the viability of F1 gametes was present. In other words, the 89 gametes represent a largely unbiased sample.

### Measuring the sporulation frequency and the clonal growth rate of F2 hybrids

Aneuploidy was not considered in the above analyses. An aneuploid gamete carries both *Sc* and *Sp* alleles for the same loci, which can mask the effects of genetic incompatibility. We found that an average of 8% of genomes contain both *Sc* and *Sp* alleles in a single gamete, so the fraction of incompatibilities that were masked in the 89 gametes is 1−0.92*^n^*, where *n* is the number of loci involved for an incompatibility. This should not bias our study because it affects fertility and viability to the same extent, but will reduce the power of detecting the different effects of incompatibility on fertility and viability, particularly when complex incompatibilities (with large *n*) are considered.

**Figure 4:**
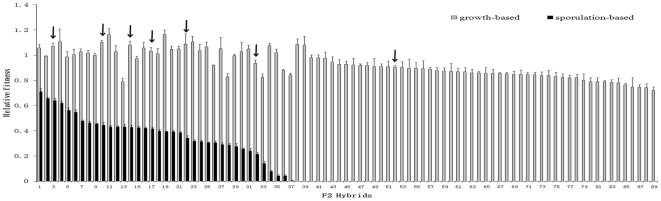
The growth-based relative fitness is significantly (*P*<10^−5^; Z-test) higher than the sporulation-based relative fitness in all 89 F2 hybrids after Bonferroni correction for multiple testing. The diploid *Sp*-YCM361 was used as the wild-type control in calculating relative fitness. Arrows point to strains that are not aneuploid. Error bar shows one standard error of the mean.

Because an intact HO gene was present in the genomes, all the 89 F1 gametes underwent autodiploidization, forming homozygous diploid F2 hybrids [22]. We confirmed experimentally the diploidy for the 89 strains. The sporulation-based relative fitness was quantified by examining the frequency of cells capable of forming tetrads in the medium for sporulation, and the growth-based relative fitness of a F2 hybrid was obtained by measuring the growth rate in YEPD, a fermentable medium both *Sc* and *Sp* having adapted to. Interestingly, the growth-based relative fitness was significantly (*P*<10^−5^; Z-test) higher than the sporulation-based relative fitness for every single F2 strain (Figure 4), when *Sp* was used as the wild-type control for calculating relative fitness. There was only one exception, if *Sc* was used as the wild-type control (data not shown). The pattern is not simply due to aneuploidy, a known factor adversely affecting cell growth [23], because the 7 strains that are not aneuploid show the same pattern (Figure 4). The average growth-based relative fitness of F2 hybrids is >0.9, suggesting sparse and weak genetic incompatibility affecting hybrid viability; in sharp contrast, the average sporulation-based relative fitness of these hybrids is <0.2, indicating prevalent and strong incompatibility affecting hybrid fertility (Figure 4). Of note, 58.4% (52/89) of the F2 strains were not able to form tetrads at all, and the underlying incompatibility is not clear, partly because the sample size is too small. We made no attempt to map incompatible genes by examining more strains, because it was not the intent of this study. We also found that strains with strong defects in tetrad formation tend to grow slowly (*R* ^2^ = 0.45, *P*<10^−12^; Pearson correlation analysis), suggesting pleiotropic effects of genetic incompatibility.

## Discussion

Our result seems to be contradictory to a previous study in which only ∼18% of the *Sc−Sp* F2 hybrids are sterile [24]. In fact, the different levels of aneuploidy of F2 hybrids between the two studies underlie the difference. The overall frequency of tetrasomy in the F2 hybrids of that study is 31%, and the number is 8.5% in our case when a similar approach was used to measure tetrasomy (244 out of 89*32 = 2848 chromosome ends show both *Sp* and *Sc* signatures) [24], which leads to the proportions of exposed incompatibilities being dramatically different between the two studies. For example, only (1–31%)∧2 = 47.6% of two-locus incompatibilities can be exposed in that study while the number is (1–8.5%)∧2 = 83.7% in our study. So, the low level of aneuploidy helped us to gain a better understanding in the effects of genetic incompatibility.

We used the sporulation frequency and the clonal growth rate at defined lab conditions as the proxies of yeast ferility and yeast viability, respectively, and observed a much higher growth-based fitness compared to sporulation-based fitness for every single F2 hybrid, strongly supporting that genetic incompatibility affects hybrid sterility more than hybrid viability in yeast. It is worth pointing out that a similar pattern was observed within the species of *Sc* [25], [26]. It should be noted that within-chromosome incompatibility [27] was not effectively investigated in this study due to the low recombination rate in the F1 hybrid. Further work is required to know if within-chromosome incompatibility evolves in a similar fashion as between-chromosome incompatibility does in affecting hybrid fitness. Using whole-chromosome replacement, a previous study examined ∼43% of the yeast genome, and identified no lethal or severe growth-related incompatibility between *Sp* and *Sc* [19]. We confirmed the previous result using a different approach, and further showed that the same is true for the rest ∼57% of the genome. When we were preparing the manuscript, a newly published paper examined about one hundred *Sc−Sp* F1 gametes using much denser markers, and came to the same conclusion that no strong two-locus incompatibility between *Sc* and *Sp* exists [28]. In principle, incompatibility involving 3 or more loci could be present. One three-locus lethal incompatibility leads to 12.5% inviable gametes, and 6 such incompatibilities are needed to cause the frequency of inviable gametes be 55.1% (1−0.875^6^), a level comparable to the observed frequency (58.4%) of complete sterility for F2 hybrids, and an even larger number of more than three-locus incompatibilities are required to generate a similar proportion of inviable gametes, which seems unlikely given the rarity of two-locus incompatibility.

The molecular mechanism underlying the finding is intriguing. A recent study revealed that *MRS1*, an *Sc* nuclear gene, is incompatible with *COX1*, an *Sp* mitochondrial gene [29]. This recessive incompatibility leads to hybrid sterility by disturbing cell metabolism on non-fermentable medium on which yeast sporulates. Interestingly, we found that one of the three major incompatibility-causing sites on the *Sc*-*MRS1* gene is polymorphic [30] (Figure S1), indicating that the derived incompatible allele is evolutionarily young. This case, together with several others found between *Saccharomyces* species [13], [29], suggests that fertility-related incompatibility often results from defects in respiration on non-fermentable media. However, despite of 58.4% of F2 hybrids in our study being completely sterile, only 13.5% (12/89) can not form colonies on the non-fermentable medium YPG (1% yeast extract, 2% peptone, and 3% glycerol) agar, indicating that defects in respiration explain only a part of the fertility-related incompatibilities.

**Figure 5:**
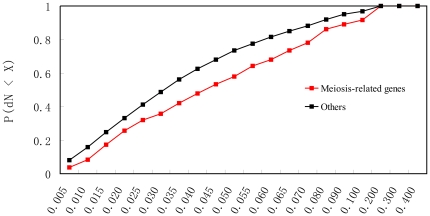
Meiosis-related genes tend to be fast-evolving compared to other genes in *Sc* (*P*<10^−3^, Mann-Whitney U test). The number of non-synonymous substitutions per non-synonymous site (dN) was calculated using *Sp* as the reference [34]. The proportion (*y*-axis) of genes with dN smaller than a certain number (*x*-axis) is shown.

Since clonal growth requires mitosis while sporulation requires meiosis, different sets of genes with different molecular features are involved in the two processes. Presumably, the chance of evolving viability-related incompatibility should be higher than that of evolving fertility-related incompatibility, because there are more genes involved in clonal growth than in sporulation. However, meiosis-related genes are generally expressed at merely detectable levels during asexual clonal growth [31], the major means of reproduction for wild yeast [32]. Recent progress in protein evolution predicts that such genes tend to evolve rapidly, because the deleterious effect of protein mis-folding caused by a non-synonymous mutation on a lowly expressed gene is relatively small [33]. Using data from our previous work [34] we found that meiosis-related genes are indeed fast evolving in the yeast (Figure 5). This may partly explain why fertility-related incompatibilities are more common than viability-related incompatibilities, and suggests a neutral view on the evolution of genetic incompatibility as well as speciation. These being said, we caution that natural populations of *Sc* and *Sp* are often found in the same habitat [22], a scenario atypical to what the Dobzhansky-Muller model is usually applied to. Also, a recent paper showed that substantial genetic incompatibility affecting both growth and sporulation can evolve between two initially-identical yeast populations after just 500 generations of independent adapted evolution in laboratory [35]. It is hard to reconcile the result with the nearly normal growth of the F1 hybrid of *Sc* and *Sp*, two species with as high as ∼15% of sequence divergence. Apparently the unique life cycle, including the fermentation-supported clonal growth and the respiration-supported sporulation, and the evolutionary history of natural *Saccharomyces* populations should be accounted for, before we can fully understand how speciation was initiated and how genetic incompatibility has evolved in yeast [22].

## Materials and Methods

### Strains and genome information

Unless otherwise stated, the F1 hybrid used in this study was made by crossing gametes of a diploid *Sc* strain YCM362 (HO, *ura3*; Y55 background) with gametes of a diploid *Sp* strain YCM361 (HO, *lys2*; N17 background), and is a gift of Dr. Calum Maclean. To reconcile our observation at chromosome 10 with a previous finding, we constructed another F1 hybrid by crossing an *Sc* strain BY4742 (MATalpha; *ho*, *his3*, *lseu2*, *lys2*, *ura3*) with an *Sp* strain YDG749 (MATa; *ho*, *ura3*); The *Sp*-YDG749 is a gift of Dr. Duncan Greig. The genome information of *Sc*-Y55 and *Sp*-N17 was obtained from a recent paper [30].

### Sample gametes of F1 hybrids

A single colony of the F1 hybrid was inoculated in 50 ml of YEPD (1% yeast extract, 2% peptone, and 2% dextrose) at 30°C until the cell concentration reached ∼1.0×10^8^ cells/ml. The culture was centrifuged, and all cells were transferred into 50 ml of medium containing 0.25% yeast extract, 0.1% dextrose, 0.82% sodium acetate, and 0.18% potassium chloride for sporulation at 30°C. One week later, an aliquot of culture was subject to microscopic examination. Cells were collected by centrifuging, washed twice using sterile water, and re-suspended in 1 ml of sterile water. 100 µl of cell suspension was diluted by adding 900 µl of sterile water, and incubated with 340 U of lyticase (Sigma) for 40 minutes at 30°C, to remove the ascus wall and release spores. The solution was then in 55°C water bath for 15 minutes to kill the remaining diploid F1 hybrids. Spores were collected, washed twice using sterile water, and plated on YEPD agar after appropriate dilution. The YEPD plates were incubated at 30°C for 4 days, single colonies were randomly picked, and streaked on fresh YEPD agar plates. For each strain, a single colony growing on the fresh plate was used for further experiments. Some of the picked strains are remaining heterozygous diploid F1 hybrids, which were identified using two markers that can discriminate *Sc* from *Sp*. The first marker (Forward: 5′-GATAGTCTCCAAAGGAAGAG; Reverse: 5′-CAATTTGGTCATTAGAAGC) is at chromosome 10, and the obtained PCR products can be cut by the restriction enzyme Xba I if the template is from *Sp*, and can not be cut if the template is from *Sc*. The second marker (Forward: 5′-GTTTCCAACCTATTCGCAA; Reverse: 5′-GCTGTATGATTGATAAAGAGG) is at chromosome 16, and the obtained PCR products can be cut by the restriction enzyme Xho I if the template is from *Sc*, and can not be cut if the template is from *Sp*. We did not use the marker separating the mating types (a/alpha versus a or alpha) of a yeast cell, because of potential autodiploidization of haploid gametes which would produce homozygous diploid F2. The two markers generated consistent results, and colonies that are heterozygous at the two loci are F1 hybrids, and thus were discarded. A total of 94 F1 gametes were obtained. Note that the identity of these gametes were further assessed with additional 93 markers covering the whole yeast genome (See below).

### Design markers for genotyping sampled gametes

A set of 93 markers were designed to genotype the 94 gametes, with approximately one marker per 100 kb of the yeast genome. For each marker, a pair of PCR primers was designed, and the PCR products derived from *Sc* and *Sp* differ in a specific restriction site (one can be cut while the other can not). For a given gamete, signatures representing both *Sc* and *Sp* can sometimes be found by a single marker, suggesting the presence of aneuploidy; in this case, the origin of this marker in this gamete was not assigned.

### Test genotype relatedness of the 94 gametes

We considered only markers at the two ends of a chromosome to ensure that they segregated largely independently, so from the 16 yeast chromosomes a total of 32 markers were used to evaluate the genotype relatedness. We used 0 and 1 to denote *Sc* origin and *Sp* origin, respectively, and did the Pearson correlation analysis for all gamete pairs (94*93/2 = 4371 pairs). A minimum number of gametes were discarded to ensure that within the retained gametes no one is significantly related to others at an FDR of 0.05/4371∼ = 10^−5^.

### Seek for two-locus genetic incompatibility

There are 4 possible combinations between two loci: *ScSc*, *ScSp*, *SpSc*, and *SpSp*; *ScSp* and *SpSc* were considered as heterogeneous combinations. Chi-square test was used to determine whether the frequency of heterogeneous combination ((*ScSp*+*SpSc*)/(*ScSc*+*ScSp*+*SpSc*+*SpSp*)) is significantly different from 0.5, the expected frequency; the frequency of heterogeneous combination was either *ScSp*/(*ScSp*+*ScSc*) or *SpSc*/(*SpSc*+*SpSp*), when one-way incompatibility was considered. We excluded 4 blocks at the left arm of chromosome 10 from analysis, and a total of 5111 block pairs were tested.

### Check the ploidy of yeast hybrids

Two pairs of PCR primers targeting the MAT locus were designed. The first pair (Forward: 5′-AGTCACATCAAGATCGTTTATGG; Reverse: 5′-ACTCCACTTCAAGTAAGAGTTTG) can produce a 544 bp fragment if the MAT locus is a; the second pair (Forward: 5′-AGTCACATCAAGATCGTTTATGG; Reverse: 5′-GCACGGAATATGGGACTACTTCG) can produce a 404 bp fragment if the MAT locus is alpha. Both of the two fragments will be observed if the cell is diploid.

### Estimate the relative fitness of F2 hybrids

Using markers that can separate the mating types (a/alpha versus a or alpha) of a yeast cell, we confirmed that all the 89 strains have already completed autodiploidization, being homozygous diploid F2 hybrids. Cells were cultured in 50 ml of YEPD at 30°C until the cell concentration reached ∼1.0×10^8^ cells/ml, and the culture was centrifuged and all cells were transferred into 50 ml of medium for sporulation at 30°C. After one week, an aliquot of culture was subject to examination of tetrad formation under microscope, and a total of 100 cells were counted to estimate the frequency (*f*) of cells having formed tetrads. Three aliquots were examined for each strain. Following the definition of relative fitness [36], the sporulation-based relative fitness (*sF*) in this work was computed using the formula:
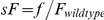
where *F_wildtype_* is the average *f* of the three examined aliquots for the wildtype strain (*Sc*-YCM362 or *Sp*-YCM361); the average *sF* of the three aliquots was used as the sporulation-based relative fitness of an F2 hybrid. For the clonal growth rate, cells were cultured in 50 ml of YEPD at 30°C for ∼18 hours, with an initial concentration of OD_660_∼ = 0.05, and the OD_660_ was measured every two hours using the 2800 UV/VIS Spectrophotometer (UNIC Corp., Shanghai). We calculated the doubling time (*t*) of cells for three consecutive periods at the logarithmic phase. Since relative fitness is defined as the average number of surviving progeny of a particular genotype compared with average number of surviving progeny of competing genotypes after a single generation [36], the growth-based relative fitness (*gF*) in this work was computed using the formula:
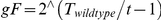
where *T_wildtype_* is the average doubling time of the three periods at the logarithmic phase for the wild-type control (the *Sc* strain YCM362 or the *Sp* strain YCM361); the average *gF* of the three periods was used as the growth-based relative fitness of an F2 hybrid.

## Supporting Information

Figure S1 The partial *MRS1* gene sequences of different *Sp* and *Sc* strains. Arrows show the non-synonymous substitutions that cause major incompatibility between *Sc-MRS1* and *Sp-COX1*. The red arrow points to the site where the incompatibility-causing mutation (A→G) is not fixed yet in *Sc* populations.
(TIF)

Table S1 (XLS)

Table S2 (XLS)
