# Supplementary figures and images for: Genetic Incompatibility Dampens Hybrid Fertility More Than Hybrid Viability: Yeast as a Case Study
Source: PLoS One. 2011 Apr 6;6(4):e18341. doi: 10.1371/journal.pone.0018341 (PMC3071822; doi:10.1371/journal.pone.0018341)

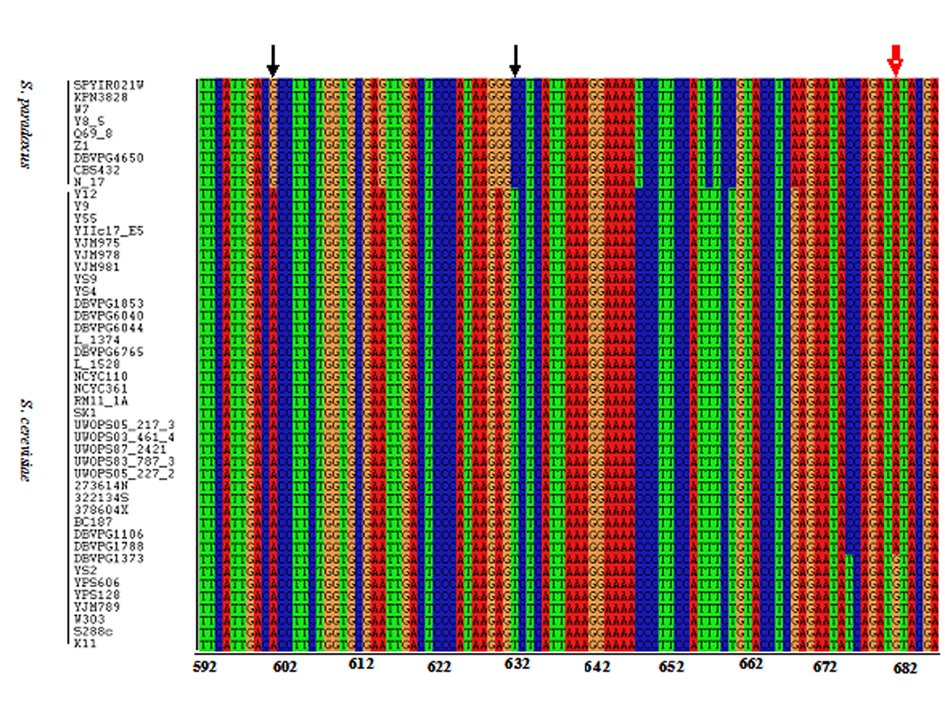

Supplement: Figure S1 — The partial MRS1 gene sequences of different Sp and Sc strains. Arrows show the non-synonymous substitutions that cause major incompatibility between Sc-MRS1 and Sp-COX1. The red arrow points to the site where the incompatibility-causing mutation (A→G) is not fixed yet in Sc populations. (TIF) [file pone.0018341.s001.tif]
